# Asymmetric pallidal neuronal activity in patients with cervical dystonia

**DOI:** 10.3389/fnsys.2014.00015

**Published:** 2014-02-11

**Authors:** Christian K. E. Moll, Edgar Galindo-Leon, Andrew Sharott, Alessandro Gulberti, Carsten Buhmann, Johannes A. Koeppen, Maxine Biermann, Tobias Bäumer, Simone Zittel, Manfred Westphal, Christian Gerloff, Wolfgang Hamel, Alexander Münchau, Andreas K. Engel

**Affiliations:** ^1^Department of Neurophysiology and Pathophysiology, University Medical Center Hamburg-EppendorfHamburg, Germany; ^2^Medical Research Council Anatomical Neuropharmacology Unit, Department of Pharmacology, University of OxfordOxford, UK; ^3^Department of Neurology, University Medical Center Hamburg-EppendorfHamburg, Germany; ^4^Department of Neurosurgery, University Medical Center Hamburg-EppendorfHamburg, Germany; ^5^Department of Paediatric and Adult Movement Disorders and Neuropsychiatry, Institute of Neurology, University Medical Center Schleswig-HolsteinLübeck, Germany

**Keywords:** cervical dystonia, GPi, GPe, microelectrode recording, LFP, oscillations, coherence, phase–amplitude coupling

## Abstract

The origin of asymmetric clinical manifestation of symptoms in patients suffering from cervical dystonia (CD) is hitherto poorly understood. Dysregulated neuronal activity in the basal ganglia has been suggested to have a role in the pathophysiology of CD. Here, we re-assessed the question to what extent relative changes occur in the direct vs. indirect basal ganglia pathway in CD, whether these circuit changes are lateralized, and how these alterations relate to CD symptoms. To this end, we recorded ongoing single cell and local field potential (LFP) activity from the external (GPe) and internal pallidal segment (GPi) of 13 CD patients undergoing microelectrode-guided stereotactic surgery for deep brain stimulation in the GPi. We compared pallidal recordings from CD patients operated under local anaesthesia (LA) with those obtained in CD patients operated under general anaesthesia (GA). In awake patients, mean GPe discharge rate (52 Hz) was lower than that of GPi (72 Hz). Mean GPi discharge ipsilateral to the side of head turning was higher than contralateral and correlated with torticollis symptom severity. Lateralized differences were absent at the level of the GPe and in recordings from patients operated under GA. Furthermore, in the GPi of CD patients there was a subpopulation of theta-oscillatory cells with unique bursting characteristics. Power and coherence of GPe– and GPi–LFPs were dominated by a theta peak and also exhibited band-specific interhemispheric differences. Strong cross-frequency coupling of low-gamma amplitude to theta phase was a feature of pallidal LFPs recorded under LA, but not GA. These results indicate that CD is associated with an asymmetric pallidal outflow. Based on the finding of symmetric neuronal discharges in the GPe, we propose that an imbalanced interhemispheric direct pathway gain may be involved in CD pathophysiology.

## Introduction

Isolated cervical dystonia (CD)—or spasmodic torticollis—is the most common form of an adult-onset, focal dystonia (Albanese et al., 2013). CD is characterized by phasic or sustained involuntary neck muscle contractions causing abnormal movements and postures of head and neck (Chan et al., 1991). The clinical presentation of many patients with CD is head turning or/and tilting to one side with rotation in the horizontal plane being the most common pattern of abnormal head and neck posture (Jankovic et al., 1991). Symptom control is often achieved with injections of botulinum toxin into overactive neck muscles (Albanese et al., 2011). For medically refractory CD, deep brain stimulation (DBS) of the internal globus pallidus (GPi) has emerged as a therapeutic option with good long-term efficacy (Krauss et al., 1999; Kiss et al., 2007; Walsh et al., 2013).

Currently, the pathophysiology of CD is not well understood. It is a widely accepted view that insufficient motor control in dystonia is critically related to functional disturbances of the basal ganglia, eventually giving rise to a systems level loss of inhibitory functions (Berardelli et al., 1998; Vitek et al., 1999; Hallett, 2011; Hendrix and Vitek, 2012). Much of the foundations for our current understanding of CD as a basal ganglia-related motor circuit disorder dates back to the 1920s when Foerster first proposed that CD symptoms would result from a deficient inhibitory striatal control of the globus pallidus (Foerster, 1921) and, more specifically, from “a focal destruction of that part of the neostriatum corresponding to the neck muscles” (Foerster, 1933). Influenced by Foerster's conceptual framework, supported by results of laboratory studies (Montanelli and Hassler, 1964) and motivated by experiences from torticollis surgery, Hassler later proposed that an unbalanced pallidal outflow could be critically involved in the asymmetrical clinical manifestation of CD (Hassler and Dieckmann, 1970).

Since then, the concepts of basal ganglia organization have markedly changed with recognition of the dichotomous organization of striatal outflow, giving rise to direct and indirect pathways through the basal ganglia, respectively, (Albin et al., 1989; Delong, 1990; Smith et al., 1998). In this influential model, the striatum exerts a dual control on basal ganglia output neurons in the GPi with opposite effects on thalamocortical circuits. Striatal direct pathway projection neurons inhibit pallidal outflow, and increased gain in this pathway is thought to facilitate movement initiation. On the other hand, an activation of indirect pathway projection neurons leads to increased activity in GPi neurons (by silencing neurons in the globus pallidus externus (GPe) which in turn leads to disinhibition of excitatory inputs to GPi from the subthalamic nucleus), thereby suppressing competing movements. In the rate model of dystonia, reduced pallidal inhibition results from a functional imbalance in the direct and indirect pathways. Both over activity in the direct or under activity along the indirect pathway, respectively, lead to excessive thalamocortical excitation and involuntary dystonic movements (Hallett, 2011). Alternatively, altered spatio-temporal patterns of neuronal activity such as excessive synchrony and/or oscillations along the different basal ganglia pathways may participate in the disruption of motor control in dystonia (Vitek, 2002). To what extent relative changes in the direct vs. indirect pathway occur in CD, whether these circuit changes are lateralized at the basal ganglia level, and how these alterations relate to CD symptoms is hitherto unclear.

There is a continuing interest in the results of single cell and local field potential (LFP) recordings from otherwise inaccessible subcortical regions of the human brain in the context of DBS surgery, as these techniques allow addressing some of the above-mentioned matters (Engel et al., 2005; Vitek et al., 2011). However, available data on lateralized neuronal activity in the GP of CD patients is sparse. Single cell recordings from the GPi of CD patients have mostly been pooled together with data from patients with phenotypically or etiologically different types of dystonias, respectively, (Starr et al., 2005; Chang et al., 2007; Weinberger et al., 2012), precluding a detailed analysis of lateralized activity changes. To our knowledge, only one study has investigated pallidal single cell activity specifically in CD patients (Tang et al., 2007). Tang et al. (2007) found no evidence for side-to-side differences in discharge rates nor patterns of GPi neurons. Several LFP studies have confirmed the presence of pronounced low-frequency oscillatory activity in the GPi of CD patients (Liu et al., 2008; Sharott et al., 2008). In a recent LFP study in the GPi of CD patients, significant interhemispheric differences in the expression of these low-frequency oscillations have been reported at the population level (Lee and Kiss, 2013). At present, it is not clear how the observed discrepancies between these single cell and LFP studies can be accounted for.

The primary goal of this study was to reinvestigate the question of lateralized differences in pallidal outflow in CD patients. To address this question, we analyzed both single cell spiking and LFP activity from GPe and GPi of CD patients undergoing microelectrode-guided DBS surgery. In the absence of control data, we compared pallidal recordings from awake CD patients with those made in a population of CD patients operated under general anaesthesia (GA), a condition under which abnormal head and neck movements or postures were absent.

## Patients and methods

### Patients

Thirteen patients (7 women, 6 men) with CD aged between 30 and 74 years were studied. The patients were referred to our hospital between February 2006 and April 2013 for stereotactic implantation of DBS electrodes in the GPi because of failure of treatment with botulinum toxin. Of these, 9 patients were operated under local anaesthesia (hereafter referred to as “LA”) and 4 patients were operated under GA due to their symptom severity or anxiety, respectively. Disease duration ranged from 3 to 30 years. The current patient sample was limited to medically refractory cases of adult onset isolated dystonia (Albanese et al., 2013). Patients with combined or complex dystonia were excluded. The majority of patients presented with a complex combination of rotation (torticollis), tilt (laterocollis), flexion (antecollis), or extension (retrocollis) of head and neck, respectively, and shoulder elevation. Rotational torticollis was the most common abnormal movement pattern. The chin was turned to the right in 6 patients (3 in the LA group), and to the left in the other 6 patients (5 in the LA group). Involuntary head posturing was accompanied by tremulous head movements in 8 patients (4 in both groups). Further demographic and clinical details of the patients are given in Table 1.

**Table 1:** **Clinical characteristics of CD patients**.

**Case**	**Gender, age at surgery**	**Disease duration (years)**	**Pattern of CD**	**Other affected regions**	**Severity of CD^§^**	**Direction of head turn**	**Head tremor**
**LOCAL ANAESTHESIA (LA)**							
1	F, 48	11	TC/LC/AC/SE	−	26	Left	−
2	F, 51	11	TC	−	19	Left	−
3	M, 45	27	TC/LC	−	15	Right	+
4	F, 30	10	TC/LC/SE	Mild WC	21	Right	−
5	F, 68	3	RC	Mild WC	17	−	+
6	F, 57	5	TC/LC/AC/SE	Mild WC	27	Left	−
7	M, 52	3	TC/LC/AC	−	26	Right	+
8	M, 50	20	LC/SE	−	21	Left	−
9	M, 72	25	TC/RC	−	n/a	Left	+
**GENERAL ANAESTHESIA (GA)**							
10	F, 42	27	TC/SE	−	17	Right	+
11	M, 49	14	TC/LC	WC	20	Right	+
12	F, 58	18	TC/LC/SE	Mild laryngeal dystonia,	22	Right	+
				tremor right hand			
13	F, 74	30	TC/LC/RC	Orofacial dystonia	22	Left	+
Mean	54 ± 12	16 ± 10			21 ± 4		

§ *As determined by the severity scale of the Toronto Western Spasmodic Torticollis Rating Scale (maximal score = 35)*.

*Abbreviations: CD, cervical dystonia; TC, torticollis; LC, laterocollis; AC, anterocollis; RC, retrocollis; SE, shoulder elevation; WC, writer's cramp; n/a, not available (quantification of patient's symptom severity not possible due to cervical plate stabilization performed in the 1980s)*.

### Clinical examination

Preoperative symptom severity was rated using the torticollis severity scale of the Toronto Western Spasmodic Torticollis Rating Scale (TWSTR, maximum score of this torticollis severity subscale = 35; Consky and Lang, 1994). Standardized videotapes were reviewed independently by two movement disorder neurologists (Simone Zittel, Maxine Biermann), and a score was given by consensus rating. Torticollis symptom severity was comparable in both patient populations (TWSTR severity score of the LA group, 21.5 ± 4.5 vs. 20.3 ± 2.4 in the GA group, Mann–Whitney test, *p* = 0.86). All intraoperative recordings and subsequent data analyses were carried out without prior knowledge of patients' symptom severity.

### DBS surgery

In all patients, bilateral DBS electrodes were implanted in a single operation. All patients had normal brain magnetic resonance imaging (MRI). As reported elsewhere in detail (Moll et al., 2009), surgical planning comprised the delineation of a reference line connecting anterior and posterior commissure in T1-weighted MRIs as well as a direct visualization of the surgical target region on MRIs fused with stereotactic computerized tomography scans for indirect and individual targeting, respectively. All procedures and collection of recordings during DBS surgery were approved by the local ethics committee and all patients gave their written and informed consent. In all patients, the posteroventral lateral aspect of the GPi was targeted 20–22 mm lateral to the midline, 2–3 mm anterior to the mid-commissural point and 3 mm below the commissural plane. Approach angles of the planned trajectories were 25 ± 11° in the sagittal and 10 ± 5° in the frontal plane. Microelectrode-guided stereotactic implantation of DBS electrodes encompassed recordings from up to five parallel tracks (mean number of recording tracks for mapping, 4 ± 1, Micro Guide, Alpha Omega Inc., Nazareth, Israel). The used recording configuration (“BenGun”) consisted of four outer tracks arranged in a concentric array around a central trajectory aiming at the target. In all but one patient (case 10), the “BenGun” was turned 45° in respect to the standard “cross-like” configuration, taking into account the elongated morphology of the globus pallidus. Typically, antero- and postero-medial trajectories were used in addition to the central electrode—in some cases together with an additional anterolateral track. Microelectrodes (Alpha Omega Neuroprobe, Alpha Omega Inc., Nazareth, Israel) were simultaneously advanced in steps of 100–500 μm. Average tip impedance was 660 ± 290 kΩ at 1000 Hz. Recordings were started 16 ± 4 mm above the radioanatomically defined target level. In the LA group, vigilance level was continuously monitored and all patients were awake and co-operative throughout the whole recording procedure. During recordings, patients were asked to lie as still as possible with their eyes closed. Only “movement-free” recordings of spontaneous activity that were made before or after the assessment of sensorimotor responses by passive or active movements, were included in the present study. In the GA group, anaesthesia was induced with an intravenous bolus of 2 mg/kg propofol. Intravenous anaesthesia was then maintained with 4.1 ± 1.3 mg/kg/h propofol in combination with 0.2 ± 0.04 μ g/kg/min remifentanil. GA patients were ventilated via an endotracheal tube with an oxygen-air mixture and anaesthetic depth and adequacy were carefully monitored throughout the whole operation by an experienced anaesthesiologist. In contrast to LA surgeries, during which some of the patients experienced involuntary muscle spasms of their neck muscles, no spontaneous movements or signs of dystonia occurred during surgery in GA.

### Data acquisition

Unit activity was bandpass-filtered between 300 and 6000 Hz, amplified (×10,000), and sampled with 24,000 Hz. Monopolar local field potentials (LFPs) were recorded from the uninsulated distal most part of the guide tube (contact size of this macrotip ~1 mm, impedance <1 kΩ), located 3 mm above the microtip. The guide tube was used as common reference. LFPs were bandpass-filtered between 1 and 300 Hz, sampled with 1.5 or 3 kHz and amplified (×5–10,000).

## Data analysis

### Spike detection and -sorting

For spike detection, a threshold was set >5 *SD* of the background noise and then adjusted to the individual signal to noise ratio of the recorded unit (Offline-Sorter, Plexon Inc., Dallas, TX, USA). Extracted total waveform length was 3 ms with a prethreshold time of 1 ms. Waveform clusters were then visualized in 2D or 3D space. Typically, plotting the first vs. second principal component of the waveform resulted in a distinct cluster in addition to a noise cluster which was discarded. In the vast majority of cases, only one single unit was recorded per electrode tip. Next, we carefully checked the autocorrelogram of the resulting spike train for the presence of a central valley, to exclude refractory period violations. Cells in which >3% of the total interspike intervals (ISI) were shorter than 2 ms and multiunit activity were excluded from this study. The signal to noise ratio was then assessed for every well-isolated neuron. To this end, the peak-to-peak amplitude of the average action potential waveform was divided by the mean of 5× *SD* of the background activity, defined as noise. Rate stability was also tested. For every unit, instantaneous firing rate was calculated as a function of time with a binning of 10 ms and visualized after smoothing using a second-order low-pass filter with zero phase shift (cut-off frequency, 10 Hz). Recordings with obvious trends or transients in the rate functions were excluded from further analysis. Only well-isolated single cell data was included in our database.

As action potential shape could help to distinguish subpopulations of pallidal neurons (Bugaysen et al., 2010), we measured a variety of spike characteristics. These measures were derived from the averaged action potential waveform of the upsampled (1 MHz) and amplitude-normalized individual spikes. This study reports peak-to-peak durations. Several basic descriptors of single neuron discharge were determined to characterize ongoing spiking activity. Mean and peak (95th percentile rate) firing rate were calculated for each neuron. To evaluate the regularity of neural discharges, we computed the coefficient of variation (CV) as the SD of the ISI distribution divided by its mean. Because the CV (ISI) may overestimate the irregularity of bursting neurons, we also used the CV2 (Holt et al., 1996)—which compensates for bursting by calculating a local CV for consecutive ISI pairs—and not for the whole spike train. We calculated the mean CV2 for each spike train. Neuronal bursting behavior was assessed by applying the Poisson burst surprise method (Legendy and Salcman, 1985). Only bursts having a surprise threshold >5 were considered, corresponding to a probability of <0.001 that the burst of interest would occur in a spike train that follows a Poisson distribution. The results of this analysis are given as the percentage of spikes that participate in those bursts. By dividing the mean ISI by the modal ISI we calculated a simple burst index for every unit, which has previously been applied to characterize human GP activity (Hutchison et al., 2003). In order to determine the time-magnitude structure of burst discharges, we calculated burst-triggered averages (Chan et al., 2011) for every unit that contained >5 bursts with a surprise value of 5. Finally, we adopted a modified version of Kaneoke and Vitek's neuronal discharge classification method (Kaneoke and Vitek, 1996) to differentiate regular, random and bursting discharge patterns (Levy et al., 2001). Briefly, discharge density histograms were created for every neuron (bin width = 1/mean firing rate) and compared with a discharge density of a Poisson process with a mean of 1 (Chi-square test). When the distribution followed a Poisson process, the neuron was classified as having a random discharge pattern. In case the distribution was not significantly Poisson, a regular or a bursting spike discharge pattern was assumed when the variance of the discharge density histogram was <1 and >1, respectively. Oscillatory properties of spike trains were assessed using the spectral method described by Rivlin-Etzion et al. (2006). Briefly, the power spectral density of a spike train was computed using nonoverlapping Hanning windows (length, 4096 ms) and a frequency resolution of 0.25 Hz. Confidence levels were created on the basis of a surrogate distribution of 100 random ISI shuffles. When peaks exceeded the 95% confidence interval, they were considered significant. Prevalence of units with significant oscillatory spiking was then assessed for each of the following frequency bands: theta (4–8 Hz), alpha (9–13 Hz), beta (14–35 Hz), and gamma (35–80 Hz).

### LFP analysis

Due to technical issues, LFPs of cases 5 (LA) and 11 (GA) were unavailable. On the basis of visual inspection, LFP recordings with exceptional noise levels and artifacts were excluded from further analysis. LFPs were then downsampled to a sampling frequency of 500 Hz and digitally band-pass filtered with a second-order notch filter at integer multiples of 50 Hz to remove line noise. To avoid phase distortions of the LFP signals, we applied zero-phase forward and reverse digital filtering. LFP power was estimated using multitaper spectral methods (time-bandwidth parameter *nw* = 3.5, 6 Slepian tapers, nfft = 500). To allow for a comparison across anaesthesia groups and to account for different LFP amplitudes depending on recording position and electrode impedance, we analyzed relative rather than absolute power. In order to obtain relative power, spectra were normalized to the total power between 1 and 250 Hz. As for the analysis of spike trains, spectra were subdivided into above mentioned frequency bands. Note that the 50 ± 2 Hz window was excluded for spectral analysis of LFPs in the gamma frequency range. Power was then averaged within these bands and significance levels of multiple *post-hoc* tests were adjusted using the Bonferroni correction.

### Spike-field coherence

Spiking activity and LFP dynamics provide complementary perspectives on neural processes (Galindo-Leon and Liu, 2010; Moran and Bar-Gad, 2010). To estimate the relation between pallidal spiking and LFPs, we calculated the spike-field coherence (SFC) as described in Fries et al. (2001). Segments of LFP signal extending ±250 ms around each individual spike were used to calculate the spike-triggered average (STA). The individual Hamming-tapered LFP-segments and the Hamming-tapered STA were Fourier transformed to calculate the corresponding power spectral densities. SFC was calculated as the ratio between the power spectrum distribution of the STA and the mean power spectral density of LFP segments. The threshold of significance was determined by randomizing the spike-times (100 repetitions) preserving the number of spikes and the ISI distribution. The mean of the randomization-based SFC plus two standard deviations was considered as the threshold of significance. Spike-LFP pairs were never from the same electrode [distance between microtip (units) and macrotip (LFPs) was 3 mm].

### Field–field coherence

The coherence *C_ij_*(*w*), defined as the measure of the linear dependency at a particular frequency *w* of two simultaneously recorded LFP signals from electrodes *i* and *j*, was calculated as:

(1) $$C_{ij}(w)=\frac{{\left|P_{ij}(w)\right|}^{2}}{P_{ii}(w)P_{ij}(w)}$$

with *P_ii_*(*w*) and *P_jj_*(*w*) the power spectral densities of channels *i* and *j*, respectively, and *P_ij_* the cross power spectral density.

### Spike–spike coherence

The frequency relation between spiking activity of two single units was estimated by the spike–spike coherence as described in Halliday et al. (1995), using the NeuroSpec toolbox (version 2.0, www.neurospec.org). Briefly, in this algorithm the discrete spiking signal (defined by the time of spikes onset in milliseconds resolution) is converted into a 1 kHz-sampled continuum signal of 0s and 1s, with 1 corresponding to a *spike* and 0 to *no-spike*. With both signals as a continuum the algorithm proceeds to evaluates their power spectral density and cross power spectral density as in Equation (1).

### Phase–amplitude coupling

Cross-frequency modulation was quantified using the previously described modulation index (Canolty et al., 2006). Briefly, LFP data was filtered into low-frequency (2 Hz-intervals between 2 and 20 Hz) and high-frequency (5 Hz-intervals between 20 and 200 Hz) bands. Analytic phase and amplitude envelope were then extracted by applying the Hilbert transform. A surrogate distribution with disrupted phase–amplitude relationship was generated by recalculating the modulation index 200 times, randomly assigning power-to-phase couples. Raw modulation indices were then transformed so that the normalized modulation index was defined as distance (in units of standard deviation) away from the mean of the distribution expected by chance (Cohen et al., 2009).

All statistical spike train and LFP analyses were carried out offline using custom written Matlab software (The MathWorks, Natick, MA). Comparisons were performed using the software Graph Pad Prism (Version 5.0, GraphPad Software, Inc., La Jolla, CA). In case of non-normal distribution of the data, non-parametric statistical testing was applied (Mann–Whitney rank sum test). Correlations were measured using Spearman's rank correlation coefficient Rho. If not stated otherwise, population averages represent data pooled from both operated hemispheres. Lateralized differences were assessed in patients with significant head turn (all patients except case #5). To allow for a calculation of meaningful averages, individual interhemispheric differences were assessed in patients with a minimum of 5 isolated single units per hemisphere. An alpha level of 0.05 was used for all statistical tests in this study. Unless otherwise noted, all values are given as mean ± *SD*.

## Results

### Neuronal data sample

We recorded the activity of 593 cells from 87 trajectories that traversed the GP region of 9 CD patients recorded under LA (*n* = 394 cells) and of 4 CD patients that were operated under GA with propofol and remifentanil (*n* = 199 cells). The average recording duration in this study was 50 s (range, 15–384). Only well-isolated single cell data was included in our database. The average fraction of ISIs that violated a 2 ms post-spike silence was 0.27 ± 0.55% and the average signal to noise ratio for the whole dataset was 2.7 ± 1.1 (noise was defined as 5 standard deviations of the background activity to comply with the peak-to-peak definition of the signal). The average signal to noise ratio in recordings carried out under GA (3.2 ± 1.4) was found to be significantly higher compared to LA recordings (2.5 ± 1.0, Mann–Whitney test, *p* < 0.0001). The incidence of isolating more than 1 single units from an individual microelectrode tip was considerably higher in GA recordings (17/189 = 9%) compared to recordings in the awake patient (6/356 = 1.7%). Possibly, this is related to reduced noise levels and neuronal firing rates in the GP of patients operated under GA (see below), but stability of recordings could also be improved under GA. We carefully combined radioanatomical and electrophysiological criteria to define 193 of the cells as GPe cells (LA, *n* = 133; GA, *n* = 60), 349 as GPi cells (LA, *n* = 220; GA, *n* = 129), and 51 as “border cells” (LA, *n* = 41; GA, *n* = 10). A zone of electrical silence and the presence of peripallidal border cells were important landmarks for the localization of white matter fascicles surrounding GPe and GPi, respectively.

### Comparison of neuronal discharges under local vs. general anaesthesia

#### GPe neurons

**Figure 1:**
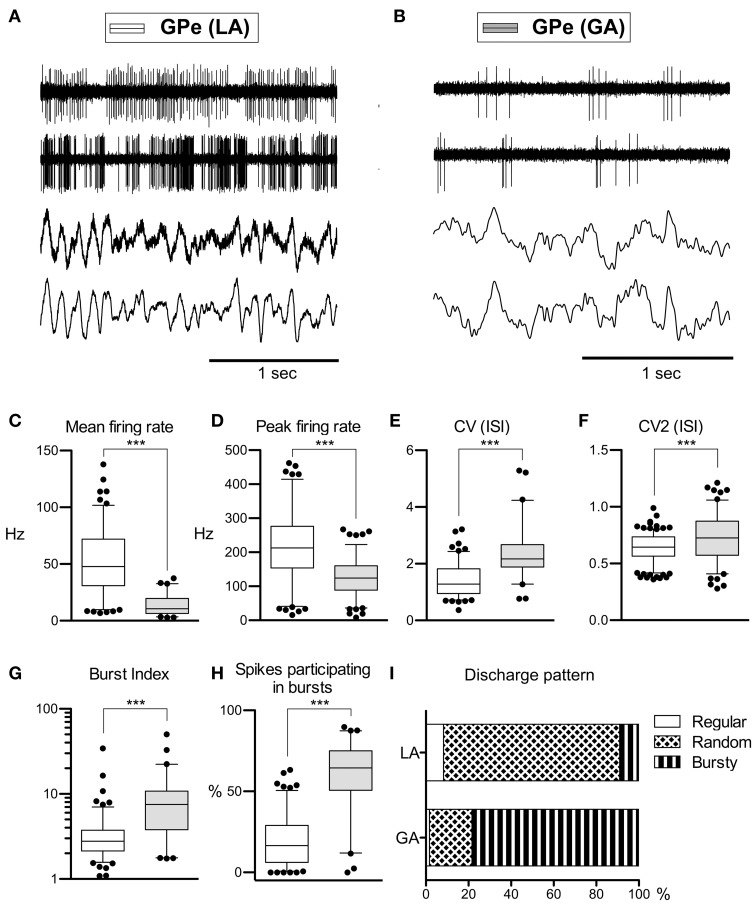
**Comparison of discharge rates and patterns of GPe neurons in CD patients under LA and GA. (A)** Raw data showing spontaneous neuronal discharges and LFPs recorded simultaneously from two different micro- and macroelectrodes in the GPe of a CD patient (surgery under LA, case 7). **(B)** Raw spike and LFP data segment recorded from the GPe of a patient operated under GA (case 13). **(C–H)** Box and whisker plots of various discharge parameters. Abbreviations: Peak firing rate, 95th percentile rate; CV (ISI), coefficient of variation of the interspike intervals; CV2 (ISI), local coefficient of variation of the interspike intervals as determined by the method of Holt et al. (1996); Burst index, mean ISI divided by the modal ISI; Spikes participating in bursts, bursts with surprise values >5 using the Poisson burst surprise method (Legendy and Salcman, 1985). Open boxes represent grand mean data from the LA group and filled boxes represent data from the GA group. Boxes extend from the 25th to 75th percentiles, whiskers represent 5th to 95th percentiles, and the median is indicated by a horizontal line. Dots represent outlying values. Asterisks denote statistical significance (^***^p < 0.001). **(I)** Stacked bar graph of GPe cells under LA and GA, depicting the relative portion of cells firing in different discharge modes as classified by the algorithm of Kaneoke and Vitek (1996).

Average peak-to-peak durations of GPe spikes were slightly but significantly longer in the LA group [Mann–Whitney test, *p* = 0.03; 260 ± 74 μs (LA) vs. 231 ± 41 μs (GA)]. Peak and average firing rates of GPe neurons in the GA group were significantly lower compared to GPe neurons recorded under LA [Mann–Whitney tests, *p* < 0.0001; peak firing rate, 219.4 ± 98.4 Hz (LA) vs. 123.8 ± 64.9 Hz (GA); mean firing rate, 52.1 ± 27.4 Hz and 13.3 ± 8.7 Hz, respectively; Table 2; Figures 1A–D]. The strong reduction in rate was accompanied by a drastic alteration of the discharge pattern. While the majority of GPe neurons (~80%) recorded under LA fired in an irregular manner, a bursty firing pattern was predominant in GPe neurons under GA. This difference was significant when comparing the proportion of neurons exhibiting different discharge modes (Chi-Square = 97.85, *df* = 2, *p* < 0.0001; Figure 1I) and for all tested descriptors of bursting behavior (Mann–Whitney test, *p* < 0.0001; e.g., 19.6 ± 15.8 vs. 59.8 ± 21.4% spikes participating in bursts, Figures 1G,H). Accordingly, both the global and local coefficients of variation had significantly higher means in the GA group compared to GPe recordings under LA (Table 2; Figures 1E,F). Burstiness of GPe neurons under LA and GA was inversely correlated with mean firing rate (LA, Spearman's rho −0.31, *p* = 0.0002; GA, Spearman's rho −0.31, *p* = 0.02).

**Table 2:** **Electrophysiological properties of GP neurons in patients with CD**.

	**GPe**		**GPi**		**Border Cells**	
	**LA (*n* = 133)**	**GA (*n* = 60)**	**LA (*n* = 220)**	**GA (*n* = 129)**	**LA (*n* = 41)**	**GA (*n* = 10)**
Firing rate (Hz)	52.1±2.4	13.3±1.1	72.0±1.9	26.0±2.0	33.0±2.3	31.7±4.5
Burst index	3.4±0.3	9.2±1.1	3.0±0.1	7.6±0.7	1.5±0.06	1.7±0.2
CV (ISI)	1.4±0.1	2.4±0.1	1.1±0.03	1.9±0.08	0.6±0.03	0.7±0.1
CV2 (ISI)	0.6±0.01	0.7±0.02	0.6±0.01	0.7±0.02	0.43±0.02	0.37±0.02
% Spikes in bursts	19.6±1.4	59.8±2.8	11.9±0.8	40.5±2.2	1.6±0.7	1.0±0.9
Peak firing rate (Hz)	219.4±8.5	123.8±8.4	277.0±6.3	208.8±11.6	86.9±8.8	71.5±9.7
P–P duration (μs)	260.4±6.5	231.1±6.1	279.5±4.5	254.5±7.2	332.2±16	320.8±17.6

*Note that results in this table are given as means ± s.e.m*.

*Abbreviations: GPe, external globus pallidus; GPi, internal globus pallidus; LA, local anaesthesia; GA, general anaesthesia; CV (ISI), coefficient of variation of the interspike intervals; CV2 (ISI), local coefficient of variation of the interspike intervals as determined by the method of Holt et al. (1996), P–P duration, peak-to-peak duration of the average action potential waveform*.

#### GPi neurons

**Figure 2:**
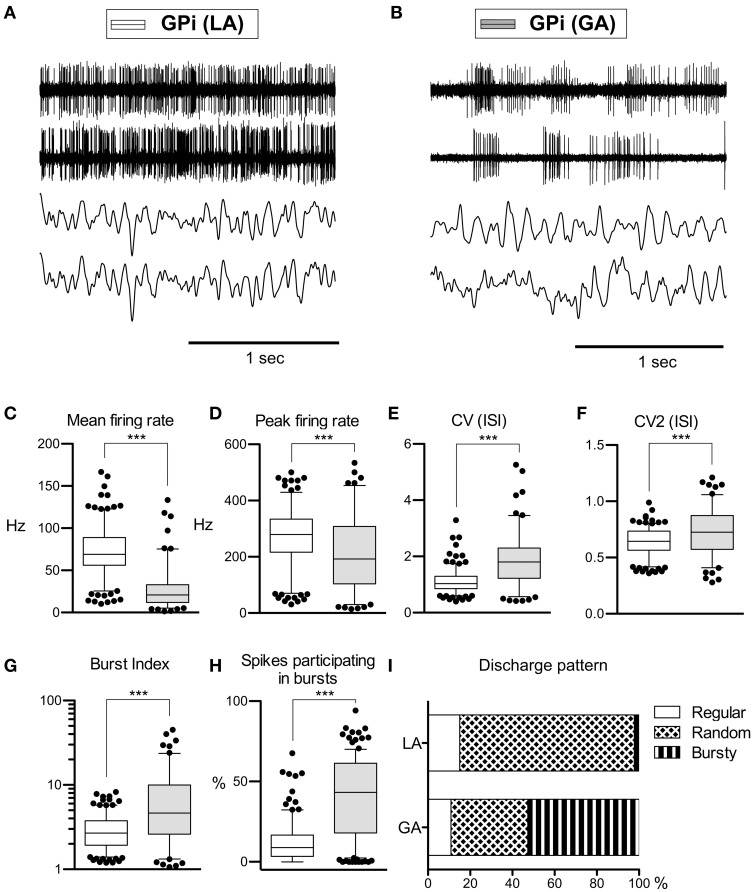
**Comparison of discharge rates and patterns of neurons under LA and GA in the GPi of CD patients. (A)** Simultaneously recorded raw spike trains and LFP data from the ventral GP of case 7 (operation under LA). **(B)** Raw traces from two representative GPi neurons recorded under GA (case 12). **(C–H)** Box and whisker plots of various discharge parameters. Conventions as in Figure 1. **(I)** Stacked bar graph of GPi cells under LA and GA, depicting the relative portion of cells firing in different discharge modes as classified by the algorithm of Kaneoke and Vitek (1996). (^***^p < 0.001).

As with GPe neurons, action potentials of GPi neurons recorded under LA were significantly longer (280 ± 66 μs) compared to waveforms derived from GA recordings (255 ± 63 μs; Mann–Whitney test, *p* < 0.001). A comparison of the raw spike trains depicted in Figures 2A,B reveals that the direction of rate- and pattern changes in the GPi associated with different anaesthesia conditions was similar to the differences seen in GPe recordings. In comparison with LA, GPi recordings under GA had significantly lower firing rates and more bursty discharge patterns (Table 2; Figures 2C–I). A significant negative correlation was noted between burstiness and mean firing rate in both anaesthesia conditions (LA, Spearman's rho −0.45, *p* < 0.0001; GA, Spearman's rho −0.24, *p* = 0.004). When comparing discharge rates between GPe and GPi within the patient group operated under LA, GPi cells (72.0 ± 28.2 Hz) fired significantly faster than GPe neurons (52.1 ± 27.4 Hz; Mann–Whitney test, *p* < 0.0001). In 5/9 patients, GPi firing rates were significantly higher compared to the GPe (Mann–Whitney tests, *p* < 0.05). In the remaining 4 patients, a non-significant trend toward higher GPi rates was observed (not shown). The average ratio of GPe:GPi firing frequency (Obeso et al., 1997) was 0.73 for recordings under LA and 0.51 for GA.

#### Border cells

**Figure 3:**
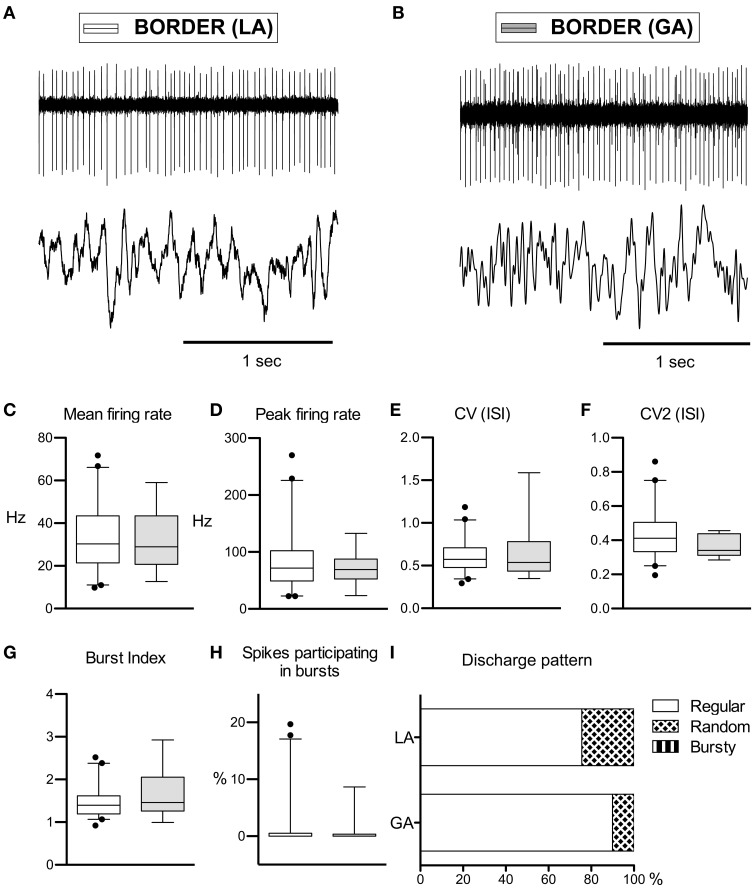
**Comparison of discharge rates and patterns of peri-pallidal border cells in patients with CD. (A)** Raw trace of a border cell recorded together with a pallidal LFP near the intersection of GPe and GPi in a patient operated under local anaesthesia (case 5). Note the highly regular firing (CV, 0.34) at intermediate frequency (mean firing rate, 26.2 Hz). **(B)** Border cell and simultaneously recorded LFP from case 10 (GA surgery). **(C–H)** Box and whisker plots of various discharge parameters. Conventions as in Figure 1. **(I)** Stacked bar graph of border cells under LA and GA, depicting the relative portion of cells firing in different discharge modes as classified by the algorithm of Kaneoke and Vitek (1996).

Peripallidal border cells were recorded at the transitions from putamen to GPe and from GPe to GPi, respectively. Occasionally, putative border cells were also found within or at the ventral border of the GPi (delineating the lamina pallidi interna and the transition to the subpallidal fiber field, respectively). In line with previous reports (Delong, 1971; Hutchison et al., 1994; Taha et al., 1997), border cells exhibited several distinguishing features compared to GP neurons. First, border cells displayed long spike durations, which, however, were not significantly influenced by the anaesthetic regime (LA, 332 ± 96 μs and GA, 321 ± 53 μs; Mann–Whitney test, *p* = 0.7). Border cells had lower peak firing rates, and generally discharged significantly slower (~30 Hz) and more regular (*CV* values <0.7) compared to both GPe and GPi neurons. Another prominent feature of border cell firing was the virtual absence of group discharges, as evidenced by low values for all measures that describe bursting behavior. In contrast to the apparent sensitivity of GPe and GPi neurons to anaesthetic drugs (see above), anaesthesia had little impact on border cell activity (Figures 3A,B). In agreement with previous observations made in generalized dystonia patients (Hutchison et al., 2003), no single descriptive parameter of neuronal activity differed significantly between LA and GA (all tests *p* > 0.05; Figures 3C–H). However, a significant difference was noted between the LA and GA group concerning the proportion of border cells that exhibited regular, random, or bursty discharge patterns (Chi-Square = 7.1, *df* = 1, *p* = 0.008; Figure 3I). The proportion of regular firing border cells was higher in the patient group recorded under GA.

### Bursting behavior

**Figure 4:**
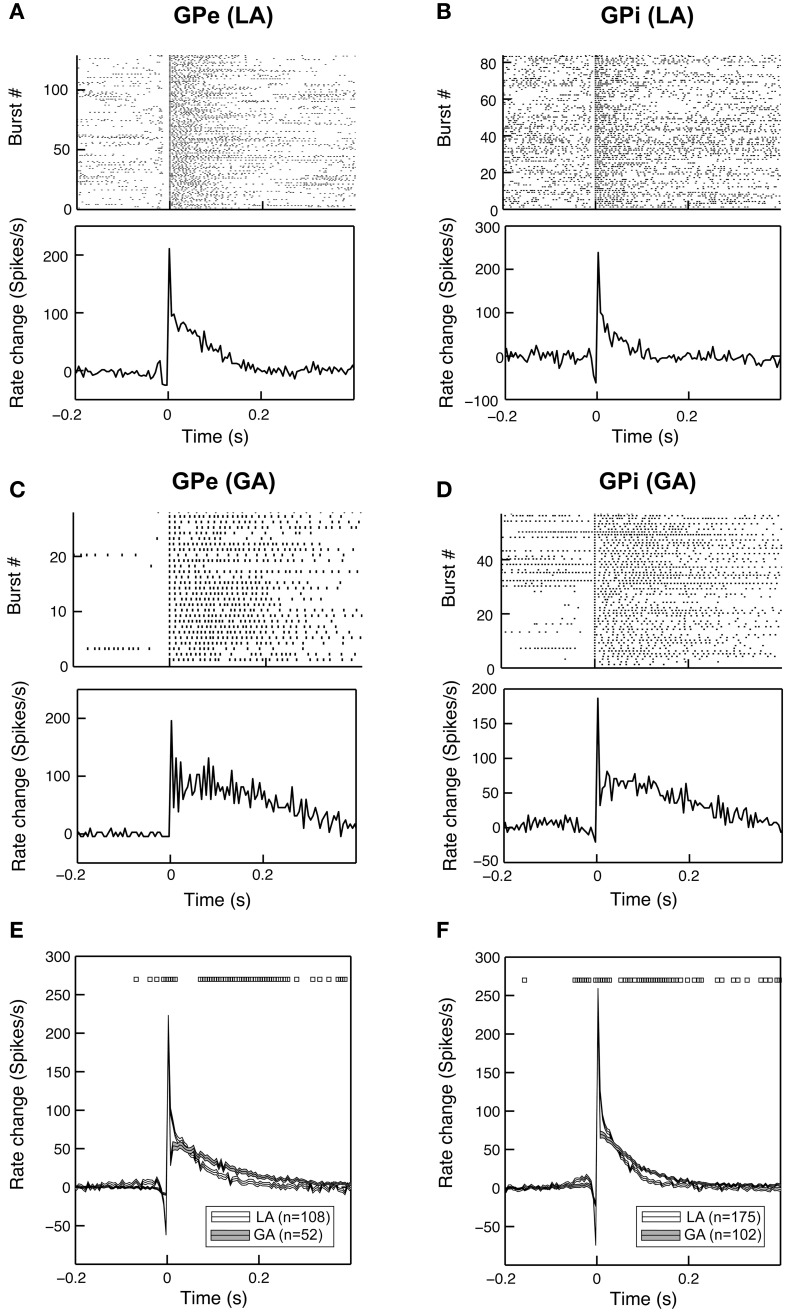
**Bursting properties of pallidal neurons recorded under GA differ from those recorded during awake surgery**. Representative examples of peri-burst analyses for neurons recorded from the GPe **(A,C)** and GPi **(B,D)** in LA and GA. The top panels depict peri-burst raster plots (aligned on burst onset). Bottom panels show mean peri-burst time histograms across all bursts following the subtraction of the cell's mean firing rate between −300 and −100 ms prior to burst onset. **(E,F)** Grand mean comparison of burst characteristics for GPe **(E)** and GPi **(F)** populations recorded under the two different anaesthesia conditions. Mean peri-burst time histograms (mean ± s.e.m.) illustrate longer burst durations under GA for both structures. The pre-burst notch is more pronounced in recordings under LA. Black rectangles above the histograms indicate regions of significance (unpaired *t*-tests, *p* < 0.05, bin-by-bin assessment of LA and GA peri-burst population averages, bin size = 5 ms).

To further characterize the time-magnitude structure of group discharges, we constructed peri-burst time histograms for all cells that contained >5 bursts. When comparing GPe and GPi group discharges within the same anaesthesia condition, burst morphology was relatively similar. The main determinants were (i) a short pre-burst decrease in discharge rate, (ii) peak intra-burst firing rates being reached around or shortly after burst onset, and (iii) a relatively rapid decay of neuronal firing rates back to baseline levels within 100–150 ms (Figures 4A,B). Bursting properties differed considerably between the two anaesthesia conditions. The pre-burst notch was absent or only moderately expressed in group discharges recorded from GPe and GPi neurons with low baseline firing rates under GA (Figures 4C,D). In both pallidal segments, average duration of burst discharges was significantly longer under GA compared to LA (Figures 4E,F).

**Figure 5:**
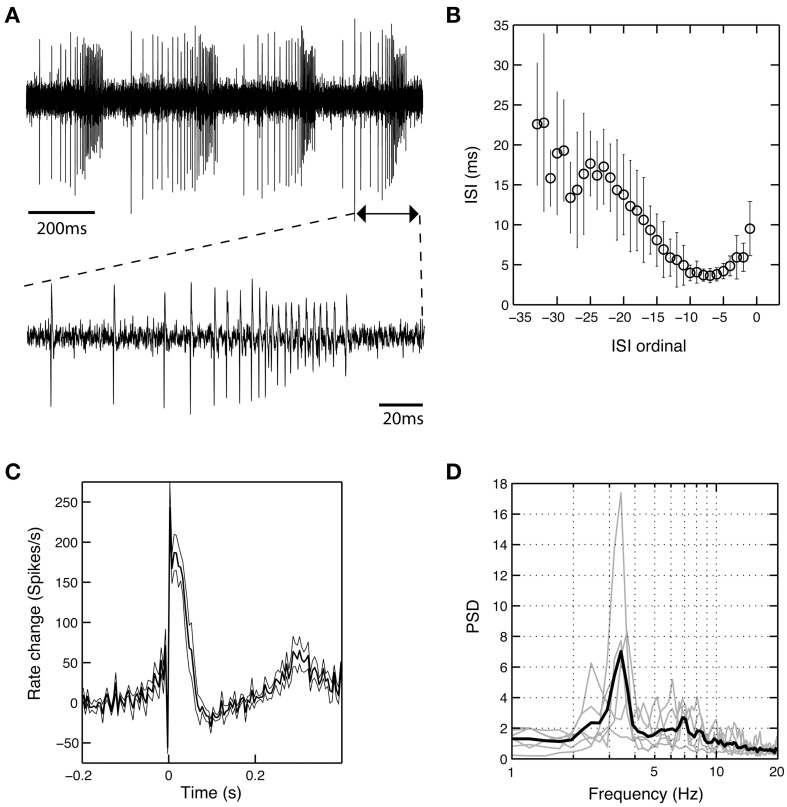
**A subset of theta-oscillatory GPi cells recorded in LA exhibited unique bursting properties. (A)** Raw trace of a neuron recorded in the GPi of case 8 (operation under LA). Note the strong periodicity, the acceleration-deceleration spiking pattern and progressive spike amplitude reduction within bursts. Sub-panel shows a single burst on an expanded time-scale. **(B)** The acceleration-deceleration pattern resulted in a parabolic-like shape when ISI duration was plotted as a function of position within the burst (ISI ordinal). Pooled data from all bursts that were recorded from the visualized neuron, with alignment at the position of the last spike in a burst. Note the reduced variability in the fine structure of the burst during the acceleration phase. Mean firing rate of this unit was 83.3 Hz and 43.8% of spikes participated in bursts with a Surprise value >5. **(C)** Population average of mean peri-burst discharge rate (±s.e.m.) for all neurons (*n* = 6, from six different patients) that exhibited the same bursting properties with an acceleration-deceleration pattern as in **(B)**. Firing rates were normalized by each neuron's average discharge rate between −300 and −100 ms before burst onset. **(D)** Mean (thick black line) and individual (gray line) normalized power spectra of the same cells as in **(C)**. All neurons had significant theta peaks in their corrected power spectra.

In six cells recorded from the GPi of six different patients operated under LA (cases 1, 2, and 5–8), we observed a unique burst discharge pattern that consisted of (i) an initial brief period of tonic single spiking turning into (ii) the actual high-frequency group discharge and (iii) a post-burst pause in neuronal firing (Figure 5A). Burst episodes were characterized by a progressive spike amplitude reduction and an acceleration-deceleration spiking pattern (see the sub-panel in Figure 5A). This acceleration-deceleration pattern resulted in a parabolic-like shape when ISI duration was plotted as a function of position within the burst (ISI ordinal). Figure 5B shows pooled data from all bursts that were recorded from the same neuron as shown in Figure 5A, with alignment at the position of the last spike in a burst. Note the reduced variability in the fine structure of the burst during the acceleration phase and the progressive lengthening of ISIs at burst end. The population average of mean peri-burst discharge rates for all neurons with this unique bursting morphology is depicted in Figure 5C. The accelerating discharge rate following burst onset and the post-burst decrease in firing rate are clearly distinguishable. Firing rate then gradually ramps up to the next peak around 300 ms, which marks the onset of the next burst and indicates strong rhythmicity in these neurons. In fact, each cell had a significant spectral peak in the lower theta-frequency range (thin gray lines in Figure 5D)—which gave rise to a distinct peak in the mean corrected power spectrum at 3–4 Hz (thick black line). The percentage of spikes participating in bursts was considerably higher in cells displaying the aforementioned bursting properties compared to the average burstiness of all GPi cells (34.7 ± 13.7% vs. 11.9 ± 11.7% for the whole sample). The average firing rate of these cells was not significantly different from the average firing rate of non-oscillatory GPi neurons recorded under LA, but lower compared to other oscillatory cells, that did not display the described unique discharge characteristics.

### Oscillatory activity in GP cells

**Figure 6:**
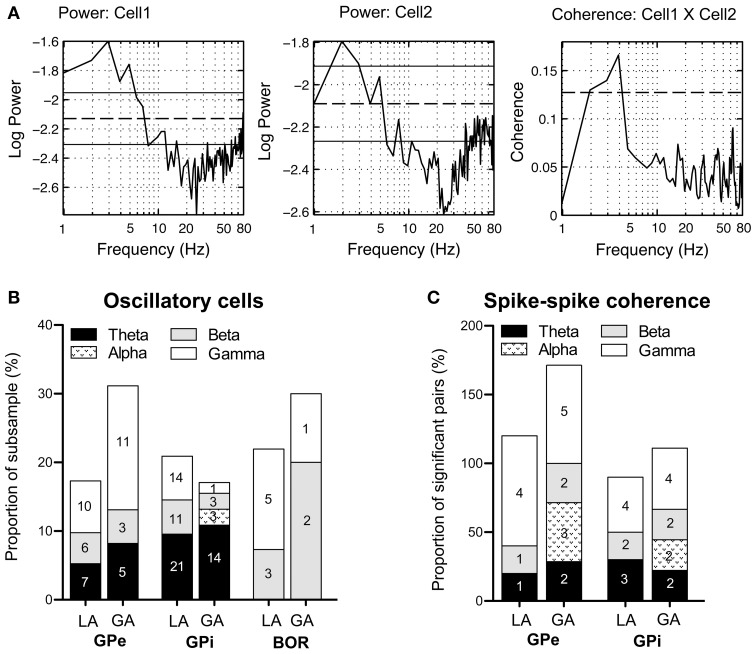
**Oscillatory properties and coherence in/between pallidal cells. (A)** Frequency domain analysis of GPi unit correlation. (Left + middle panel) Log plots of estimated power spectra for two simultaneously recorded cells from the GPi of case 3 (awake surgery). Dashed horizontal lines represent the asymptotic value of each estimate and solid horizontal lines give the estimated upper and lower 95% confidence limits. (Right panel) Coherence estimate. The horizontal dashed line gives the estimated upper 95% confidence limit. **(B)** Stacked bar graph illustrating absolute numbers and relative portions of cells (relative to the whole subsample) with significant spectral peaks in their corrected power spectra for each frequency band. **(C)** The stacked bar graph depicts for each frequency band number of cell pairs with significant spike–spike coherence and percentage relative to all significantly coherent pairs. Individual pairs of simultaneously recorded cells could have more than one significant peak in their coherence spectra. Therefore, the sum of all relative portions may exceed 100% for a given subsample.

As rhythmic neuronal activity in the GP has repeatedly been proposed to have a role in the pathophysiology of dystonia, we also assessed spike-train periodicity. Figure 6A (left and middle panel) shows two cells recorded from the GPi of case 3 (operation under LA), that exhibited prominent oscillatory activity as evidenced by spectral peaks in the theta-frequency range. The relative proportion of cells with significant peaks in their corrected power spectra was about the same for all investigated cell types and patient groups (GPi/LA, 20%; GPe/LA, 15%; GPi/GA, 16.3%; GPe/GA, 26.3%; Border cells/LA, 19.5%; Border cells/GA, 20%). The stacked-bar graph in Figure 6B provides their percent abundance relative to all neurons within a patient subsample for each frequency band, as well as numbers of cells contributing to each portion. Irrespective of anaesthesia conditions, theta-oscillatory neurons constituted the largest fraction of cells with significant spectral peaks within the GPi. Gamma-oscillatory units in the GPe were noticeably more prevalent under GA. In fact, strong oscillatory modulations at high frequencies were repeatedly observed in the peri-burst time histograms of these cells (see Figure 4C). Significant spectral peaks of border cells originated from their rhythmic single cell spiking (see Figures 3A,B), with peak frequencies roughly corresponding to their average discharge rates. In both the LA and GA sample, the average firing rate of GPi oscillatory neurons was significantly higher compared to cells without significant peaks in their corrected power spectra (GPi/LA, 80.4 ± 30.2 Hz vs. 69.9 ± 27.3 Hz, *t*-test, *p* = 0.03; GPi/GA, 39.6 ± 33.8 Hz vs. 23.2 ± 18.4 Hz, Mann–Whitney test, *p* = 0.02). In the GPe, however, average discharge rates of oscillatory and non-oscillatory cells were not significantly different (GPe/LA, 50.8 ± 31.9 Hz vs. 52.4 ± 26.7 Hz, *t*-test, *p* = 0.81; GPe/GA, 15.3 ± 10.6 Hz vs. 12.6 ± 7.9 Hz, Mann–Whitney test, *p* = 0.46). No significant differences were noticed in any sample between the burstiness of oscillatory cells and their non-oscillatory counterparts (Mann–Whitney tests, all *p*-values > 0.05).

### Spike–spike coherence

Pairwise coherence was investigated in a total of 126 simultaneously recorded spike train pairs. The majority of pallidal unit pairs exhibited flat coherence spectra and cross-correlations. The percentage of GPi cell pairs with significant peaks in the coherence-spectra was not significantly different between LA (10/41, 21.3%) and GA recordings (9/38, 23.7%, Fisher's exact test, *p* = 0.74). However, the proportion of significantly coherent cell pairs in the GPe was significantly lower in LA (5/26, 19.2%) than in GA recordings (7/21, 33.3%, Fisher's exact test, *p* = 0.04). Irrespective of anaesthesia and pallidal structure, significant peaks in the spike–spike coherence spectra consistently occurred in the theta-, beta-, and gamma frequency range. Figure 6A (right panel) shows significant theta-coherence in a cell pair that was simultaneously recorded in the GPi of case 3 and Figure 6C provides numbers and relative portions of significant coherence spectra between pallidal neurons for each frequency band. Alpha-band coherence was restricted to recordings under GA and related to propofol-associated alpha-spindling (not shown). Spike–spike coherence in the gamma-band range was more prevalent in GPe than in GPi.

### LFP power

**Figure 7:**
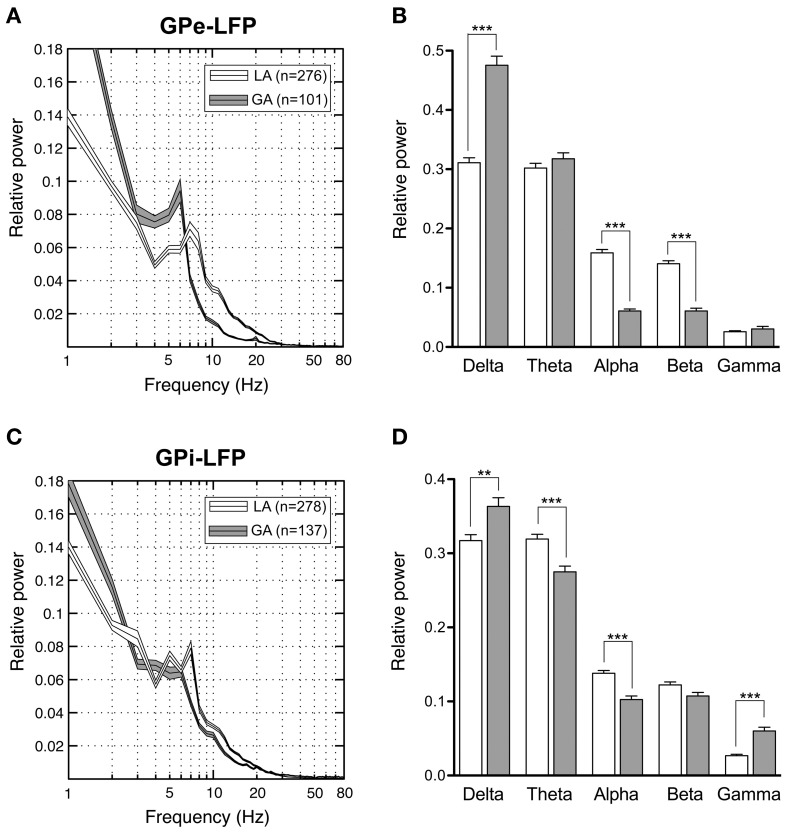
**Power spectral analysis of pallidal LFPs in CD patients. (A,C)** Grand averages of relative LFP power spectra (mean ± s.e.m.), normalized to total power between 1 and 250 Hz, that were recorded under different anaesthetic modalities from GPe **(A)** and GPi **(C)**. Depicted frequency range is 1–80 Hz. **(B,D)** Frequency-band specific comparisons of relative LFP power between LA (open bars) and GA (filled bars) in GPe **(B)** and GPi **(D)**. Asterisks indicate significant differences (^**^p < 0.01; ^***^p < 0.001 after Bonferroni correction).

Our pallidal LFP database of patients operated under LA comprised artifact-free recordings from >250 sites in 8 patients (average number of LFP recording sites per patient: 35 ± 23) located within GPi and GPe, respectively. Power spectra of these recordings were compared to those derived from >100 LFP recordings under GA in 3 patients (average number of LFP recording sites per patient: 40 ± 27) from each pallidal structure (Figure 7). Power spectra of both GPe and GPi recordings under LA were dominated by a peak in the theta-frequency range (peak frequency ~7 Hz). As Figures 7A,C illustrate, LFP power under GA also peaked in the theta range in both pallidal subdivisions, albeit with higher peak power and a peak frequency shift toward lower frequencies. When meaning all GPi–LFPs for individual patients, each patient displayed a main low-frequency peak between 2 and 8 Hz in the power spectrum (not shown). A second distinct spectral peak in the alpha—frequency range was recognizable in 7 out of 9 pallida of patients operated under LA. The GA group generally exhibited significantly more delta but less alpha power compared to LA in both GPe and GPi (Figures 7B,D). Due to a more pronounced peak frequency shift of GPe spectral power under GA, beta but not theta power was significantly lower compared to GPe–LFP power under LA. In contrast, GPi theta power was significantly higher in awake patients compared to patients operated under GA. When comparing the spectral profile of GPe vs. GPi within the LA patient group, significant power differences were found in two frequency ranges. While theta LFP power was significantly higher within GPi (Mann–Whitney test, Bonferroni-corrected *p* < 0.0001), alpha power was significantly higher within GPe (Mann–Whitney test, *p* = 0.02 after Bonferroni correction).

### LFP–LFP coherence

**Figure 8:**
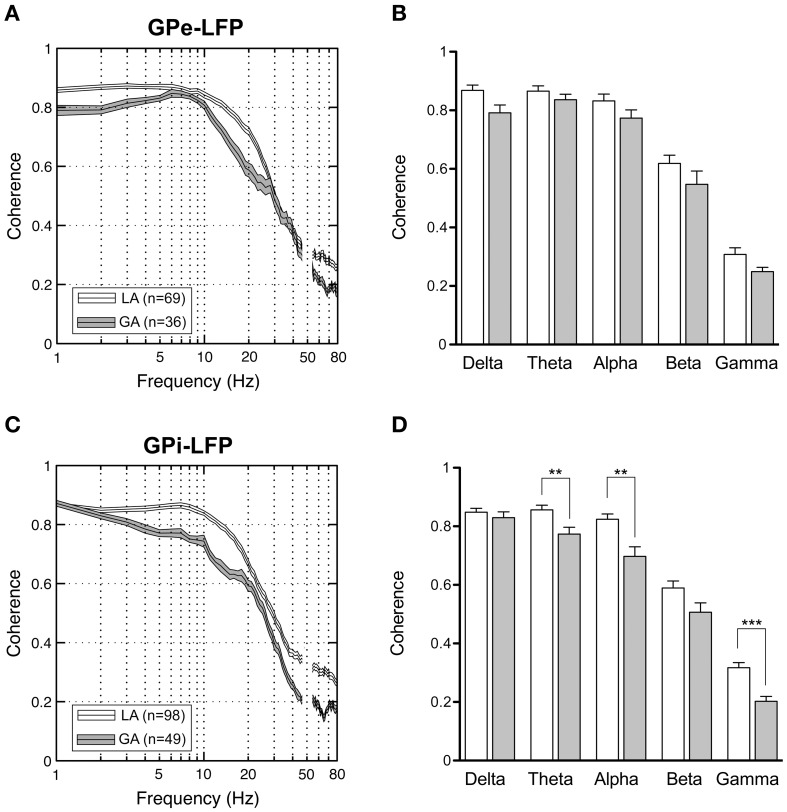
**LFP–LFP coherence analysis of pallidal LFPs**. For each pair of LFP recordings from GPe and GPi of patients operated under LA and GA, respectively, coherence was computed and averaged. **(A,C)** Grand averages of coherence spectra (mean ± s.e.m.) for GPe **(A)** and GPi **(B)** recordings (frequency range, 1–80 Hz). Highest coherence was selective to frequencies below 10 Hz. **(B,D)** Frequency-band specific comparisons of mean LFP–LFP coherence between LA (open bars) and GA (filled bars) in GPe **(B)** and GPi **(D)**. Asterisks denote comparisons where statistical significance was reached (^**^p < 0.01; ^***^p < 0.001 after Bonferroni correction).

When comparing simultaneously recorded sites within GPi and GPe, respectively, peak LFP-coherence (with values >0.7) consistently occurred at low frequencies <10 Hz. The magnitude of LFP coherence under GA was generally lower compared to LA across the whole frequency spectrum in both pallidal structures (Figures 8A,C). However, when band-specific LFP–LFP coherence was assessed statistically, significant differences between the two anaesthesia conditions were confined to the region of the GPi. Here, theta-, alpha-, and gamma coherence were significantly higher for LA compared to GA recordings (Figure 8D; Mann–Whitney tests, Bonferroni-corrected *p*-values = 0.003, 0.005, and <0.001, respectively). In contrast, no significant differences in LFP coherence between LA and GA were observed within GPe (Figure 8B). When comparing the magnitude of LFP–LFP coherence between GPe and GPi in patients operated under LA, no significant differences were observed in any frequency band. The only significant difference in LFP coherence under GA was detected in the gamma frequency range, which was significantly higher in GPe compared to GPi (Mann–Whitney test, *p*-value after Bonferroni-correction = 0.004).

### Phase–amplitude coupling in pallidal LFPs

**Figure 9:**
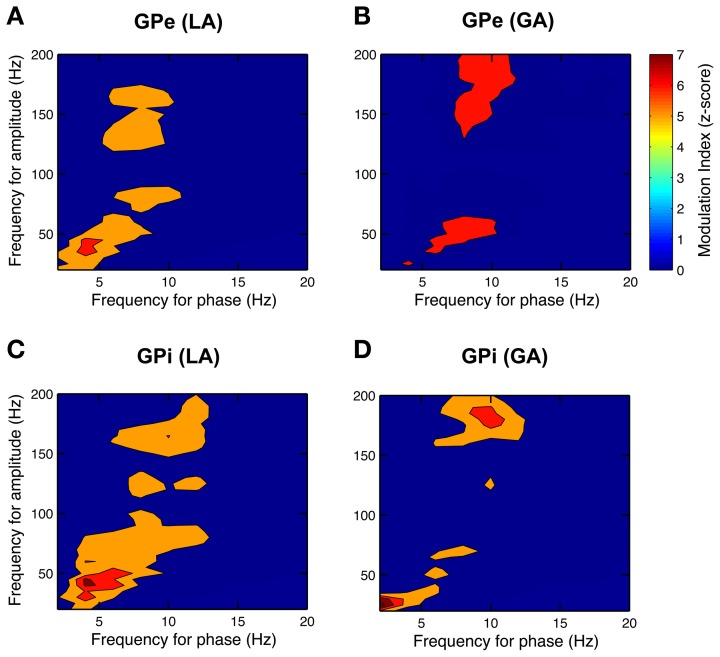
**Phase–amplitude coupling in the GP of CD patients differs between LA and GA**. All panels depict grand averages across subjects and show values of *z*-scored modulation indices that reached statistical significance (Bonferroni-corrected *z*-score > 4). Co-modulograms of both GPe– **(A)** and GPi–LFPs **(C)** under LA revealed strong cross-frequency coupling between theta phase and amplitude in the lower gamma band. Under GA, this theta–gamma co-modulation was shifted toward higher frequencies for both phase and amplitude in the GPe **(B)** and was completely absent in the GPi **(D)**. A significant cross-frequency interaction between alpha-phase and amplitude in the higher gamma range was observed in both structures and under both anaesthetic modalities.

After having confirmed the presence of a dominant theta-rhythm in both power and coherence of pallidal LFPs, we wanted to know whether low-frequency phase and high-frequency amplitude of different LFP rhythms are coupled. To this end, we used the method of Canolty et al. (2006) and scanned a broad range of phase–amplitude pairs—each created by extracting phase information from a lower and amplitude from a higher frequency band—for the presence of significant co-modulation. The pseudocolor plots in Figure 9 depict grand averages of *z*-scored modulation indices across all patient populations and pallidal substructures, respectively. Only statistically significant values (Bonferroni-corrected *z*-score > 4) of cross-frequency phase–amplitude coupling are shown. Our analysis revealed especially strong theta-phase modulation (peaking at 3–7 Hz) of the lower gamma-band (peaking at ~40 Hz) in the GPi, but also in the GPe of patients operated under LA. Moreover, high gamma amplitude (>125 Hz) was modulated by the phase of oscillatory activity in the upper theta/lower alpha frequency range. Interestingly, the latter theta/alpha-to-high-gamma comodulation was also observed in both GPe and GPi in recordings under GA. In contrast, theta-low gamma coupling was noticeably less expressed or absent in the GPi under GA. Instead, we observed substantial delta-phase modulation of oscillations in the beta-frequency (20–30 Hz) range. No difference in the phase–amplitude coupling pattern was found between ipsi- and contralateral recordings for any structure or condition.

### Spike-field coherence

SFC between LFPs and pallidal single unit activity was generally small and tended to disappear in the relatively high variance of the estimate. Estimating phase-coupling between spikes and LFPs with the pairwise phase consistency method of Vinck et al. (2010) produced essentially the same results, indicating that local spiking activity is not locked to global pallidal population oscillations picked up at distant sites.

### Correlation of electrophysiological recordings with demographic and clinical variables

**Figure 10:**
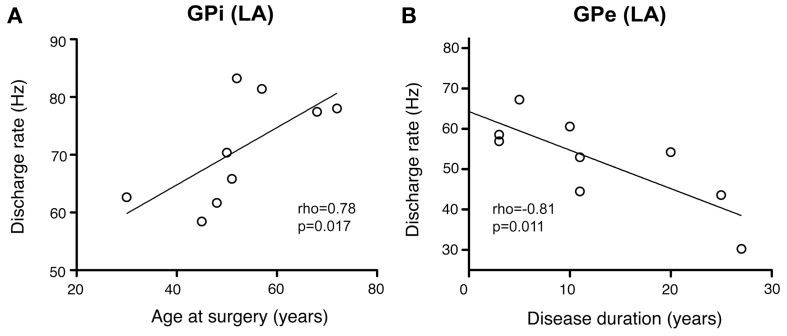
**Scatterplots of pallidal discharge rates as a function of demographic and clinical variables**. Data points in both graphs represent mean firing rates for each patient (pooled from recordings in both hemispheres). **(A)** Average firing rate of GPi neurons showed a significant positive correlation with mean age at time of DBS surgery. **(B)** Mean GPe discharge rates were correlated inversely with disease duration.

As illustrated in Figure 10A, a significant positive correlation was observed (Spearman's rho 0.78, *p* = 0.017) between patient's age and internal pallidal firing rate (both hemispheres pooled). Age and GPe discharge rate were not correlated (Spearman's rho 0.12, *p* = 0.776). A significant negative correlation was observed between disease duration and GPi firing rate (Spearman's rho −0.7, *p* = 0.043; not shown). However, an even stronger negative correlation was found between disease duration and external pallidal discharge rate (Spearman's rho −0.81, *p* = 0.011; Figure 10B). We then wanted to test whether the proportion of lifetime encumbered with CD also correlated with pallidal discharge rates. Therefore, we normalized the duration of CD symptoms to age at surgery by calculating the ratio: CD duration/age at surgery (Lumsden et al., 2013). While the correlation of this ratio and GPe rate missed statistical significance (Spearman's rho −0.62, *p* = 0.085), a significant negative correlation with internal pallidal discharge rate was found (Spearman's rho −0.72, *p* = 0.037). No significant correlation was found between patient's age and disease duration (Spearman's rho −0.29, *p* = 0.46) or torticollis symptom severity, respectively, (Spearman's rho −0.07, *p* = 0.843).

### Lateralized differences

In patients with surgery in LA, from 3 to 22 GPi neurons were isolated per hemisphere (mean, 12 ± 5 neurons) and 5 or more units were obtained in 16/18 hemispheres. This allowed us to perform meaningful individual comparisons of discharge parameters between the two sides in most patients.

**Figure 11:**
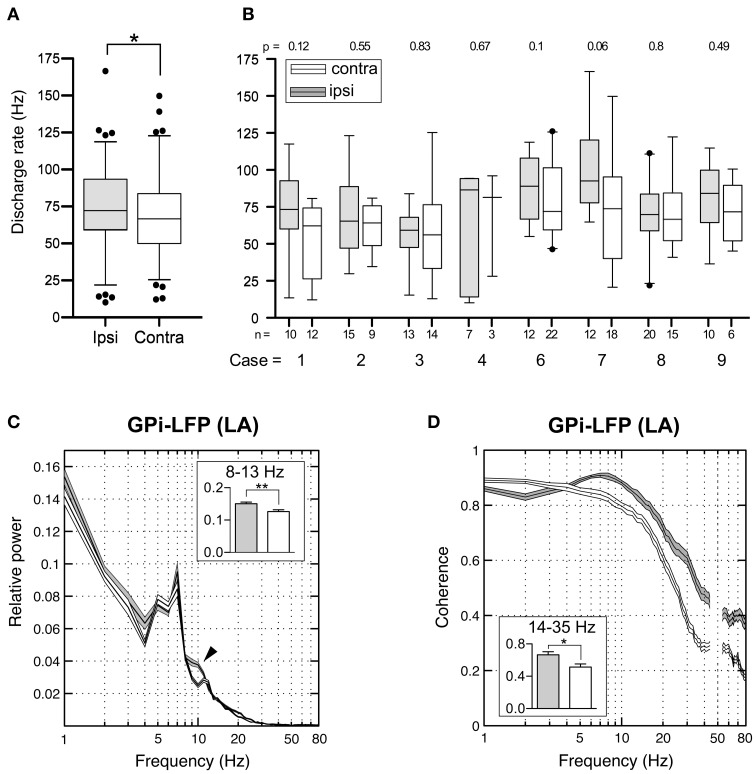
**Interhemispheric differences in single cells and LFPs of the GPi recorded during awake surgery. (A)** At the population level, mean firing rates of GPi neurons recorded ipsilateral (filled boxes) to the side of head turn were significantly higher compared to cells recorded from the contralateral hemisphere (open boxes). **(B)** When compared individually for each patient, this difference did not reach statistical significance. **(C)** Average relative power spectra (mean ± s.e.m., frequency range 1–80 Hz) of LFPs recorded in the GPi ipsilateral (gray, *n* = 108) and contralateral (white, *n* = 111) to the direction of head turn (patients operated under LA). Note the bihemispheric expression of a prominent peak in the theta-frequency range. Relative alpha power is significantly higher in ipsilateral pallidal LFPs (arrowhead). Inset: Statistical comparison of ipsi- and contralateral LFP power between 8 and 13 Hz. (**D**) Side-specific coherence spectrum of LFP pairs that were simultaneously recorded in the GPi of awake patients. Comparison of ipsilateral (gray, *n* = 38) and contralateral (white, *n* = 39) GPi–LFP pairs. Inset: Ipsilateral GPi–LFP coherence was significantly higher in the beta frequency range. Asterisks refer to significant differences (^*^p < 0.05; ^**^p < 0.01, Bonferroni corrected).

To better understand the relation between pallidal outflow and the asymmetric clinical manifestation of CD, we assessed possible lateralized differences in relation to the direction of head turn (chin direction) in patients with torticollis. In the 8 patients of the LA group with significant head turn, the population average of GPi discharge rates ipsilateral to the side of head turn (*n* = 99) was significantly higher compared to the contralateral side (74.8 ± 27.3 vs. 67.9 ± 27.5 Hz (*n* = 99), Mann–Whitney test, *p* = 0.04, Figure 11A). When this comparison was done for each patient individually, it was noted that ipsilateral discharge rates were always slightly higher than contralateral, but lateralized differences only reached statistical trend level in 3/8 patients (Figure 11B). In order to control for spurious side-to-side differences, we also performed a comparison between neurons recorded in the left (*n* = 120) and right (*n* = 100) GPi, respectively. Average discharge rates did not differ (unpaired *t*-test, *p* = 0.7). When the interhemispheric comparison was done for GPi cells recorded from anaesthetized patients, no significant difference in firing rate was found between the two sides [ipsilateral (*n* = 53) 25.6 ± 19 vs. contralateral (*n* = 76) 26.2 ± 24.4 Hz; Mann–Whitney test, *p* = 0.9]. Having observed that side-to-side difference were present under LA, but not GA, we then wanted to test whether these lateralized differences are a feature of the whole pallidal axis, including the GPe. Notably, when comparing mean firing rates of GPe neurons ipsilateral (*n* = 54) and contralateral (*n* = 79) to chin direction in the LA group, no significant interhemispheric difference was found (Mann–Whitney test, *p* = 0.5)—suggesting that the described discharge asymmetry in our patients was confined to the level of basal ganglia output neurons in the GPi. Peak firing rate and descriptors of firing patterns showed no side-to-side difference, neither in the LA group (Mann–Whitney tests; peak firing rate, *p* = 0.3; CV (ISI), *p* = 0.1; CV2 (ISI), *p* = 0.5; participation of spikes in bursts, *p* = 0.4; Burst index, *p* = 0.9) nor in patients with GA. Likewise, no significant difference in any other GPe discharge parameter was found between the two sides. In patients with significant head turning that were operated under LA, the number of GPi neurons with significant spectral peaks showed no lateralized difference (ipsilateral *n* = 26/contralateral *n* = 28). Because the presence of dystonic head tremor could influence pallidal neuronal activity (Raz et al., 2000), we wondered whether tremor-related inhomogenieties in our patient sample could partially explain some of our results. Noticeably, the mean neuronal firing rate of GPi cells between tremulous vs. non-tremulous CD patients operated under LA was not significantly different (Mann–Whitney test; *p* = 0.6).

Subsequently, we addressed the question whether similar lateralized differences might be observed in LFPs recorded from the GPi. The power spectral profiles of LFPs recorded from hemispheres ipsilateral and contralateral to the side of head turning were largely similar and both dominated by a distinct peak in the 4–9 Hz range (Figure 11C). After Bonferroni correction for multiple comparisons, significant band-specific differences were found for two frequency ranges: While alpha-power was significantly higher in the ipsilateral GPi (Mann–Whitney test, *p* = 0.0028), power in the gamma-frequency range was significantly higher in the GPi contralateral to the side of chin direction (Mann–Whitney test, *p* < 0.0001). Side-specific assessment of LFP–LFP coherence revealed strongly coherent GPi–LFP signals in both hemispheres across a broad range of frequencies. Beta-band LFP coherence within the GPi ipsilateral to the side of chin rotation was significantly higher compared to the opposite side (Figure 11D; Mann–Whitney test, Bonferroni-corrected *p*-value = 0.04).

### Correlation with disease severity (TWSTR severity score)

**Figure 12:**
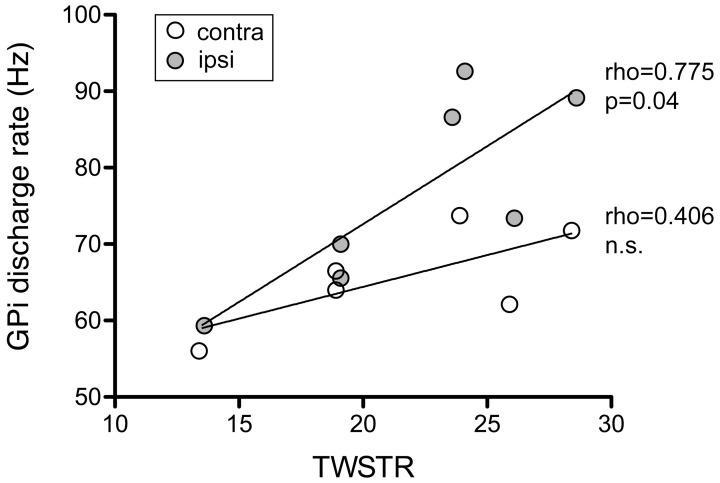
**Ipsilateral, but not contralateral, GPi firing rates of awake patients showed a significant positive correlation with torticollis symptom severity**. Each data point represents collapsed averaged firing rates from the GPi contralateral (open circles) and ipsilateral to head turn (filled circles) for each patient. Torticollis severity was assessed using the severity subscale of the Toronto Western Spasmodic Torticollis Rating Scale (maximum score = 35).

Mean discharge rate of GPi cells pooled from both hemispheres was not correlated with torticollis severity (Spearman's rho 0.37, *p* = 0.4). Noticeably, for neurons recorded from the GPi ipsilateral to head turn, there was a significant correlation with the severity of dystonic symptoms (Figure 12; Spearman's rho 0.775, *p* = 0.04). In contrast, no significant relationship was found between contralateral GPi firing rate and TWSTR severity score (Spearman's rho 0.406, *p* = 0.4).

## Discussion

The principal findings of the current study were that lateralized differences of mean GPi discharge rates—depending on the direction of head excursion—exist in patients with CD. Mean GPi discharge ipsilateral to the side of head turning was higher than contralateral and correlated with torticollis symptom severity. Lateralized differences were absent in recordings from patients operated under GA. Mean GPe discharge rate was lower than in GPi and was inversely correlated with disease duration. Another key finding of our study was that the GPi of CD patients comprised a subpopulation of theta-oscillatory cells with unique bursting characteristics. Power and coherence of ongoing GPe and GPi LFPs were dominated by a theta peak and also exhibited band-specific interhemispheric differences. Cross-frequency coupling of low-gamma amplitude to theta phase was especially pronounced in pallidal LFPs recorded under LA. Finally, single cell spiking was not locked to global pallidal LFP oscillations recorded at distant sites.

### Firing rates in GPe and GPi

Previous studies in CD (Tang et al., 2007) or other forms of dystonia (Merello et al., 2004; Starr et al., 2005) have reported parity in discharge rates between pallidal compartments, corresponding to a calculated ratio of GPe:GPi firing frequency (Obeso et al., 1997) close to 1. This ratio was 0.73 in our study, which is in-between the generally lower values (~0.6) reported for this ratio in PD (Starr et al., 2005; Tang et al., 2007) and GPe:GPi firing frequency ratios of ~0.8–1 in recordings from healthy primates (Starr et al., 2005; Elias et al., 2008; Erez et al., 2011).

In our study, the mean firing rate of GPi neurons pooled from both hemispheres of awake CD patients was 72 Hz. This is in excellent agreement with the mean rate of 71 Hz that was reported by the only available study investigating pallidal discharges in a cohort of CD patients (Tang et al., 2007). Thus, internal pallidal discharge rates of CD patients are grosso modo similar to those being reported for healthy nonhuman primates (Filion and Tremblay, 1991; Wichmann et al., 2002; Starr et al., 2005; Elias et al., 2008; Erez et al., 2011) but considerably higher compared to the generally low GPi rates reported for patients with other types of dystonia, in particular generalized forms (Vitek et al., 1999; Sanghera et al., 2003; Merello et al., 2004; Starr et al., 2005; Zittel et al., 2009).

Contrasting with the report of Tang et al. (2007), firing rates of neurons in the GPe (mean rate, 52 Hz) in our study were significantly lower than in GPi but also lower compared to the mean rates of ~65 Hz that are typically found in the GPe of healthy monkeys (Wichmann et al., 2002; Starr et al., 2005; Elias et al., 2008; Erez et al., 2011). Instead, average GPe rates were in the same range as values reported for patients suffering from Parkinson's disease (Favre et al., 1999; Starr et al., 2005; Tang et al., 2007). Consistent with the finding of higher than normal firing rates of STN neurons in patients with CD and segmental dystonia (Schrock et al., 2009), this finding suggests that—similar to PD—the indirect pathway is also overactive in CD. Taken together, our finding of decreased firing rates in GPe—but close to normal rates in GPi—could be explained by dual hyperactivation of both the direct and indirect pathways in our CD patients, which is also in accordance with a current model of dystonia (Vitek, 2002): While excess direct pathway gain will *decrease* GPi activity, overactivation of the indirect pathway will *increase* GPi activity (via weakened inhibitory inputs from GPe and disinhibited excitatory projections from STN). The net result may be no change in GPi activity.

### Influence of anaesthetic modality on pallidal activity in CD

It has long been known that pallidal neuronal activity in patients with dystonia is severely affected by propofol (Hutchison et al., 2003), which is the most frequently used anaesthetic in surgery for movement disorders. However, these observations were mainly made in patients suffering from generalized dystonia, where GA is frequently employed due to severity of symptoms. Our results confirm the notion of a propofol-related reduction in pallidal firing rates in conjunction with profound discharge pattern changes toward slow non-oscillatory bursting, and extend it to a homogeneous patient sample with a focal manifestation of dystonia. To the best of our knowledge, firing rates and -patterns of pallidal border cells have hitherto not been reported in patients with CD. Unlike pallidal neurons, discharge parameters of border cells showed no differences between LA and GA and were comparable to recordings from healthy primates (Bezard et al., 2001). Both observations are consistent with previous research in patients with generalized dystonia (Hutchison et al., 2003), suggesting that spontaneous border cell activity is unaffected in different types of dystonia and not strongly affected by propofol anaesthesia. The reported differences in firing rates and bursting characteristics between the two anaesthetic modalities may aid future microelectrode-guided mappings of the pallidal region in DBS surgery for CD, especially when performed under GA with propofol and remifentanil.

### Correlation of pallidal discharge parameters with clinical variables

In addition to these findings, we found a series of significant correlations of pallidal discharge characteristics with clinical and demographic variables in our dataset, which have not been previously reported. In our dataset, patient's age was positively correlated with internal pallidal discharge rates of awake CD patients. This may be of interest because neuronal activity of dystonia patients is typically compared to data derived from Parkinson's disease patients (Silberstein et al., 2003; Starr et al., 2005; Tang et al., 2007), a patient group that is on average considerably older. Thus, inferences made based on differences in neuronal firing rate between different patient groups should take this possible bias into account. GPi and GPe firing rates were both negatively correlated with disease duration, suggesting that the dual hyperactivation of direct and indirect pathways (Vitek et al., 1999) is strongest in patients with a long history of CD symptoms.

Age at surgery and disease duration have repeatedly been identified as predictors of surgical outcome in patients with generalized dystonia treated with GPi–DBS (Isaias et al., 2008; Valldeoriola et al., 2010). However, data regarding predictors of surgical outcome in patients with CD are conflicting. While one study reported better surgical outcomes in patients with shorter disease duration (Yamada et al., 2013), another found no evidence for any such correlation (Witt et al., 2013). We also observed a significant negative correlation between the proportion of life lived with CD and firing rates in the GPi. Future studies should test whether response to pallidal DBS in CD depends on this measure, as has been demonstrated for childhood dystonia (Lumsden et al., 2013).

### Lateralized differences

Perhaps the most surprising observation of the present study was the finding of significant interhemispheric differences of pallidal outflow in relation to the clinically determined head excursions. The discharge rate of neurons located within the GPi ipsilateral to the side of head turn were significantly higher compared to their contralateral counterpart. This asymmetry was absent under GA and not observed in the GPe of awake patients. In conjunction with lines of evidence from our rate analysis in GPe and GPi (see above), our finding of imbalanced GPi rates suggests that on top of a symmetrical overactivation of indirect pathways, an asymmetric overactivation of the direct pathway activities may play a role in CD. How could this firing rate asymmetry be significant to the pathophysiology of CD? Lateralized inhibitory outflow from the GPi would lead to unbalanced activation of thalamo-cortical modules and/or brain stem centers and result in uncontrollable activation of neck muscles on one side—thus, contributing to the asymmetric clinical manifestation of CD. While the observed absolute side-to-side difference was moderate (~7 Hz), network modeling studies have suggested that comparably small firing rate changes in a population of noisy or irregularly firing neurons may translate into larger changes in the activity of downstream neurons (Adair, 2001).

However, lateralized differences in individual patients reached non-significant trend levels at best, suggesting that large numbers of recordings may be required to detect this firing rate difference at the single-subject level. In conjunction with a possible undersampling of the neck-representing pallidal territory that is involved in CD, this might also explain differences between our findings and that of a previous investigation on pallidal physiology reporting the absence of side differences in CD patients (Tang et al., 2007).

A possible link between imbalanced pallidal outflow and the asymmetric manifestation of CD symptoms might also influence the surgical approach, e.g., the selection of the side for DBS in unilateral interventions or staged bilateral procedures. The question of therapeutic dominance of one GPi is undecided (Walsh et al., 2013). There are conflicting reports of beneficial effects on CD symptoms of GPi surgery both ipsilateral (Islekel et al., 1999; Moll et al., 2008; Torres et al., 2010) and contralateral (Kavaklis et al., 1992; Escamilla-Sevilla et al., 2002) to the side of head deviation. Our finding of a significant correlation between ipsi- but not contralateral GPi discharge rates and torticollis symptom severity may provide support for the view that the striato-pallidal system with less over activity along the direct pathway (i.e., the GPi ipsilateral to the direction of head turn) could be an important determinant in the pathophysiology of CD.

It is noteworthy that, in contrast to bursting or oscillation spike train properties, ipsilateral GPi discharge rate was the only neuronal activity parameter that correlated significantly with torticollis severity in our study. This is in agreement with a similar observation from a pallidal physiology study in patients with phenotypically and etiologically diverse types of isolated dystonia (Starr et al., 2005) and suggests that information relevant to disease severity in dystonia may primarily be encoded in rate, rather than pattern of pallidal discharges. However, it is important to note that Starr et al. (2005) found an inverse relationship between GPi rate and baseline dystonia severity score, while GPi rates were positively correlated with severity of neck affection in our CD patients, as assessed by a focal dystonia rating scale (TWSTRS).

### Single cell oscillations and coherence of neuron pairs

The proportion of significantly oscillatory cells (15–30%) in our study is in the same range as previously reported for patients with different types of isolated dystonia (Starr et al., 2005), but contrasts with a lower incidence of occurrence reported for CD patients (Tang et al., 2007). Single cell oscillations in the theta frequency range were consistently found in both pallidal divisions and under both anaesthetic modalities, which is in line with previous observations reporting pronounced low-frequency oscillatory activity in pallidal neurons of awake dystonia patients (Starr et al., 2005). In addition, we observed significant oscillatory activity of neuronal discharges in the beta- and gamma- frequency range. Higher frequency oscillations were particularly expressed within burst firing of GPe neurons under GA. Furthermore, our study provides evidence for the existence of oscillatory long-range synchrony between distant (~2 mm) pallidal neurons, which—to the best of our knowledge—has not been reported before (Tang et al., 2007). Interestingly, both oscillatory single cell firing and coherence in the alpha- frequency range was restricted to recordings under GA. This suggests a frequency-specific modulation of neuronal activity related to anaesthesia, such as propofol- related spindling.

### GPi high frequency bursters in CD oscillate at theta- frequencies

A subpopulation of neurons in the GPi of patients with Parkinson's disease exhibiting a unique type of neuronal discharge was first described by Taha et al. (1997), who coined the term “GPi tonic-burster” for units combining discharge characteristics of two presumably different cell types that are regularly encountered in recordings from the rodent and primate GPe (Delong, 1971; Bugaysen et al., 2010; Benhamou et al., 2012). On the one hand, these cells share a tonic level of high frequency discharges with neurons called high frequency pausers (HF-P). On the other hand, some specific burst characteristics of GPi tonic bursters (high intraburst frequencies, progressive spike amplitude reduction) are similar to those of low-frequency bursters (LF-B). In conjunction with other lines of evidence (Taha et al., 1997; Chan et al., 2011), our results suggest that one striking feature of GPi tonic bursters is their robust rhythmicity. Taking this into account and to comply with the classic terminology introduced by DeLong (Delong, 1971), we propose that these cells may be termed oscillatory high-frequency bursters (OHF-B). All recorded OHF-B neurons had a unique burst structure. Each burst had a stereotypical acceleration-deceleration pattern, i.e., intraburst ISI reached a minimum midway through and became progressively longer toward the end of each burst. Together with the periodic transitions between bursting and quiescence, this peculiar discharge characteristic is reminiscent of “parabolic bursting” (Ermentrout and Kopell, 1986), referring to the parabola-like appearance ISI sequences in a burst (for an example, see Figure 5B). It is interesting to note that the peak oscillation frequencies of OHF-B discharges in CD and PD are markedly different. While OHF-B cells in CD patients peaked in the theta range, their oscillation frequency in patients suffering from Parkinson's disease was confined to the alpha- frequency range (Chan et al., 2011). In any case, oscillation periods of individual OHF-B cells were remarkably robust. Although sparsely distributed, it is therefore, conceivable that OHF-B cells represent a distinct class of pacemaker-neurons in the GPi. Their rhythmic bursting may be involved in the generation and/or maintenance of a steady network rhythm in larger neuronal populations, similar to “hub neurons” orchestrating widespread synchronization in developing networks (Bonifazi et al., 2009). It is of interest that only two of the patients where OHF-B cells were recorded, had head tremor. Therefore, this set of neurons is unlikely to represent true tremor cells (which drive head tremor or are driven by proprioceptive feedback from tremulous muscle movements). Moreover, it is unlikely that OHF-B properties are related to injury discharge or other recording instabilities because the average recording duration of stable activity from these cells was 79 ± 55 s.

### Low-frequency rhythmicity in pallidal local field potentials

In our study, GPi–LFPs of awake CD patients had dominant spectral components at low frequencies (<10 Hz), adding to the existing evidence that increased oscillatory activity in the extended theta/alpha- frequency range is a prominent feature of LFPs recorded in the GPi of patients with different types of dystonia (Silberstein et al., 2003; Chen et al., 2006b; Liu et al., 2006), including CD (Lee and Kiss, 2013).

To our knowledge GPe–LFPs have not been reported in CD. Our data show that strong low-frequency oscillations also are cardinal features of LFPs recorded from the GPe in CD patients. Furthermore, this study also extends the observation of higher theta power in GPi compared to GPe, as has previously been reported for patients with various types of isolated dystonia (Chen et al., 2006b), to a group of homogeneous cases in terms of clinical phenotype and localization of dystonic symptoms. Our finding of higher alpha power in the GPe compared to GPi has not been reported previously—perhaps due to the common usage of relatively broad spectral frequency ranges (typically, 4–10 and 11–30 Hz) in many studies investigating LFPs in dystonia (Silberstein et al., 2003; Lee and Kiss, 2013).

Pallidal low-frequency LFP oscillations have been demonstrated to be correlated with (Chen et al., 2006a) and coherent to (Liu et al., 2006) dystonic muscle activity. Involuntary dystonic muscle spasms in turn have been shown to be synchronized by a descending oscillatory drive at similar frequencies in patients with isolated dystonia (Tijssen et al., 2000) and directional analysis has suggested a causal link between pallidal LFP oscillations <10 Hz and dystonic muscle activity (Sharott et al., 2008). In order to address the question whether low-frequency oscillations in the pallidal LFPs are relevant to the expression of clinical head excursion, we recorded from CD patients that were operated under GA and showed no signs of dystonic muscle activity. Rather unexpectedly, we also observed distinct theta peaks in power spectra of pallidal LFPs recorded from these fully anaesthetized and symptom-free patients. This suggests that low-frequency rhythmicity seems to represent a general characteristic of pallidal LFP activity in CD that does not simply arise as a consequence of dystonic muscle activity. It is noteworthy in this context that coherence in two distinct frequency ranges of GPi–LFPs was observed to be significantly higher in the awake state compared to LFPs recorded under GA. In addition to elevated levels of low-frequency coherence in the theta- and alpha- band, GPi–LFP coherence in the gamma- band was also higher in patients operated under LA. Higher intrapallidal coherence levels could have either have a generative role for dystonic muscle contractions (which was not specifically assessed in our study) or arise as a consequence of proprioceptive feedback from overactive neck muscles.

Side differences in GPi LFP power between 4 and 30 Hz have recently been reported in patients with CD (Lee and Kiss, 2013). The authors found a slight, yet non-significant preponderance of higher 4–30 Hz-power in the GPi ipsilateral to the direction of head turning. Supporting the notion that the direction of head excursion in CD is related to interhemispheric differences of pallidal neuronal activity, we found significantly higher alpha- band power and beta- band coherence of GPi–LFPs ipsilateral to the direction of head rotation compared to the contralateral hemisphere.

### Pallidal theta–gamma phase–amplitude coupling

Another novel finding in our study is the demonstration of strong phase–amplitude cross-frequency coupling between theta/alpha and gamma oscillations in pallidal LFPs. Theta/alpha-gamma coupling was noticeably less prevalent in LFPs recordings under GA, despite the presence of strong oscillatory activity in the LFPs. In conjunction with previous lines of evidence showing a relationship between pallidal gamma-band activity and the scaling of ongoing movements (Brucke et al., 2012), it is therefore, conceivable that the frequently observed excessive theta/alpha oscillations in GPi–LFPs of dystonia patients influence aspects of involuntary muscle contractions via modulation of activity in the gamma-frequency range.

Spurious coupling in EEG or LFP frequency comodulation measures have been demonstrated to result from art factual sharp edges in the data (Kramer et al., 2008). This is a serious concern for studies in patients with hyperkinetic movement disorders, as movement artifacts could produce abrupt voltage changes, eventually resulting in artificial frequency comodulation results. In our study, head fixation precluded major head movements resulting from CD symptoms. Furthermore, the analysis of phase–amplitude measures was restricted to periods of stable recording conditions and the absence of head movements, respectively, as confirmed by concurrent stationary unit activity.

### Pallidal units are not locked to globally coherent LFP oscillations

Previous attempts to link pallidal single neuron activity to the LFP in patients with dystonia have reported a prevalence of significant spike-field locking between 10% (Weinberger et al., 2012) and 30% (Chen et al., 2006b). In the study of Weinberger et al. (2012), the prevalence of units with significant SFC was ~8% in unit and LFP recordings from the same electrode. This proportion dropped to 2% when the relationship of unit discharges to LFPs recorded from a second microelectrode (located 600 μm apart) was investigated. Together with the lack of a consistent relationship between spikes and LFPs (recorded 3 mm away from the microtip) in our study, this distance-related decay profile of SFC may indicate that ongoing oscillatory activities in the GP of CD patients—although highly coherent at the LFP level—does not necessarily couple spiking output to input signals on a global scale. Furthermore, the generally smaller SFC values for single unit activity compared to that for multiunit activity may have contributed to this null finding (Zeitler et al., 2006). Contrasting with the absence of long-range SFC within the GP, however, a subset of neuronal pairs showed significant spike–spike coherence. The presence of such long-range interactions at the level of single pallidal neurons is suggestive of an alternative explanation that takes into account the functional topography of the GP. In this respect, the low SFC may be due to the sampling of different pallidal subterritories, as electrode contacts for unit and LFP recordings with an interelectrode distance of 3 mm were arranged along the dorso-ventral pallidal axis. In contrast, coherence estimates between pairs of spikes or LFPs were based on recordings in the horizontal plane (2 mm away) that were most likely situated in similar functional divisions of the GP.

### Limitations of the approach

One of the major limitations with invasive recordings from patients undergoing DBS surgery is the absence of data from a healthy control group. Data obtained from the diseased brains of human patients (collected during therapeutic interventions) are commonly compared to similar recordings from the healthy nonhuman primate. Conclusions from these comparisons across species must necessarily remain tentative and should be interpreted carefully.

Furthermore, fixation of the patient's head and neck in an overcorrected position may have provoked compensatory neck muscle activity. Thus, it is impossible to distinguish whether our results obtained from awake CD patients were the cause or the consequence of dystonic symptoms (Berardelli et al., 1998). While it is common practice to compare the results of invasive recordings between different movement disorders (Silberstein et al., 2003; Starr et al., 2005; Tang et al., 2007), we chose a different approach and compared data from awake CD patients with recordings in patients with the same diagnosis, but DBS surgery under GA, which was associated with complete suppression of all dystonic muscle activities.

Torticollis severity cannot be directly assessed in the context of head fixation during conventional frame-based stereotactic interventions. It would therefore be interesting to see how our results compare to pallidal recordings in patients without head fixation undergoing frameless stereotactic DBS surgery.

Being based on microelectrode-guidance, our study does not suffer from limitations relating to imprecise localization of recording sites that are inevitably associated with postoperative LFP recordings using the permanently implanted DBS electrode. Nevertheless, it is important to note that a definite localization of recording sites, as obtained by, e.g., histological control in animal studies, is impossible in studies of invasive recordings in humans.

When microelectrode-guided DBS surgery in the same patient is performed on different days—e.g., in the context of a staged procedure—lateralized differences of neuronal activity may be difficult to assess, as mere implantation of a DBS electrode can exert significant effects on patient's symptoms (Cersosimo et al., 2009). Such patient-to-patient variations were minimized by only including patients receiving bilateral DBS-implants on the same day.

Although the present study contains the hitherto largest number of neurons recorded from the GP of CD patients, the overall number of patients included in our study was relatively small due to a limited number of CD cases undergoing DBS surgery. In the light of conflicting evidence from a previous study (Tang et al., 2007), there is clearly a need for larger-scale studies with higher statistical power to clarify the issue of possible lateralized differences in pallidal outflow in patients with CD.

## Conclusion

Taken together, our results provide tentative support for the possibility that CD may be associated with a symmetrical indirect-pathway overactivity in conjunction with imbalanced interhemispheric activity levels along the direct pathway. The direct pathway ipsilateral to the direction of head turning could, according to this view, fail to compensate for excess indirect pathway activity. We hypothesize that asymmetrical pallido-thalamic output gain could then arise as a consequence of a disrupted balance of direct and indirect pathway overactivation on one side and be an important factor contributing to the asymmetric manifestation of CD symptoms.

### Conflict of interest statement

The authors declare that the research was conducted in the absence of any commercial or financial relationships that could be construed as a potential conflict of interest.
